# Association of pregnancy outcomes with neonatal TSH levels in euthyroid singleton pregnancies

**DOI:** 10.3389/fendo.2025.1508532

**Published:** 2025-05-29

**Authors:** Weiwei Feng, Mengfan Song, Yu Meng

**Affiliations:** ^1^ Department of Obstetrics and Gynecology, International Peace Maternity and Child Health Hospital, School of Medicine, Shanghai Jiao Tong University, Shanghai, China; ^2^ Shanghai Key Laboratory of Embryo Original Diseases, Shanghai, China

**Keywords:** pregnancy outcomes, neonatal screening, thyroid function, FT4, thyroid stimulating hormone

## Abstract

**Background:**

The association between pregnancy outcomes and neonatal TSH levels remains controversial. The aim of this study is to explore the association between pregnancy outcomes and neonatal TSH to interpret neonatal TSH indicators reasonably.

**Methods:**

This study was a large-sample, retrospective observational analysis conducted at a tertiary hospital in Shanghai. Data from 52,027 pregnant women who underwent routine prenatal examinations at the International Peace Maternity and Child Health Hospital from January 2013 to December 2016 were extracted from the electronic medical record system. The normal reference ranges for TSH and FT4 were established based on the 95% confidence interval of the total study population, and singleton pregnant women with normal thyroid function were selected for analysis. Non-parametric Mann-Whitney U test and Chi-square test were used to assess the relationship between pregnancy outcomes and neonatal TSH levels. A restricted cubic spline Cox regression model was used to evaluate the nonlinear association between various pregnancy outcomes and neonatal TSH. Univariate and multivariate logistic regression analyses were conducted to adjust for confounding factors and further analyze the relationship between pregnancy outcomes and elevated neonatal TSH levels.

**Results:**

A total of 24079 pregnant women were included in this study. In univariate analysis, neonatal TSH levels were significantly higher in women with advanced maternal age and in multiparas (P < 0.05). Women with preeclampsia and cesarean section, neonates with fetal distress, male neonates, macrosomia, LBW and premature births had a significant increase in neonatal TSH level (P < 0.05). After adjusting for confounding factors, multivariate regression analysis showed that the following pregnancy outcomes remained strongly associated with neonatal TSH elevation (P<0.05): preeclampsia (OR=2.238, 95% CI 1.454 ~ 3.446), cesarean section (OR=1.404, 95% CI 1.179 ~ 1.672), advanced maternal age (OR=1.322, 95% CI 1.012 ~ 1.728), and preterm birth (OR=2.408, 95% CI 1.683 ~ 3.445).

**Conclusion:**

Neonatal TSH levels are influenced by factors such as advanced maternal age, preeclampsia, cesarean delivery, and preterm birth, which can lead to elevated TSH in newborns.

## Introduction

Thyroid-stimulating hormone (TSH) levels are the primary screening indicator for identifying congenital hypothyroidism (CH) in newborns. CH is one of the most common and preventable causes of neonatal intellectual disability worldwide, with an incidence rate of approximately 1 in 2,500 to 1 in 4,000 live births (1). Since the clinical presentation of CH at birth is often subtle, early screening is considered a cost-effective clinical practice (2), as timely thyroid hormone replacement therapy can prevent brain damage (3). In most developed countries, screening for CH has become mandatory. However, despite the significant public health value of neonatal TSH screening, false-positive results due to transient neonatal hyperthyrotropinemia are not uncommon. Studies have shown that various maternal and fetal factors during pregnancy and delivery can influence neonatal TSH levels (4, 5), potentially leading to transient neonatal hyperthyrotropinemia, which complicates the interpretation of screening results. Among these influencing factors, maternal characteristics, especially demographic factors such as age, parity, and obesity, have received relatively little attention. Additionally, the impact of various pregnancy complications, such as intrauterine growth restriction (6), preeclampsia (7), and gestational diabetes mellitus (GDM) (7), on neonatal TSH levels warrants consideration. Given the complexity of pregnancy and delivery, careful interpretation is required when assessing the association between these factors and neonatal thyroid hormone levels. In conclusion, understanding the influence of maternal and fetal factors on neonatal TSH concentrations is critical for the accurate interpretation of neonatal TSH screening results. This study aims to elucidate the impact of various maternal and fetal factors during pregnancy and delivery on neonatal TSH levels, to facilitate better interpretation of neonatal TSH screening outcomes.

## Methods

### Subjects

This study is a retrospective cohort analysis conducted at a tertiary specialized hospital in Shanghai. We extracted medical records of 52,027 pregnant women who underwent prenatal examinations at the hospital between January 2013 and December 2016 from the electronic medical record system, selecting 24,079 eligible participants. The study received approval from the hospital’s ethics committee and adhered to approved guidelines. Exclusion criteria were as follows: (1) a history of pre-pregnancy thyroid disease; (2) multiple pregnancy; (3) conception by ART; (4) use of medications impacting thyroid function; (5) incomplete or missing FT4, TSH, and TPOAb tests in the first or third trimester; (6) absence of neonatal heel blood TSH test results; (7) abnormal FT4 and TSH levels in the first or third trimester; (8) delivery outside the study hospital.

### Data collection

Serum samples were collected from the median cubital vein in fasting conditions during early pregnancy (11–13 weeks) and late pregnancy (32–34 weeks). Within 6 hours of collection, the serum was separated by centrifugation. Following the manufacturer’s protocol, concentrations of TSH, FT4, and TPOAb were measured using the Architect i2000 immunoassay analyzer (Abbott Laboratories, Chicago, USA). Neonatal heel blood TSH screening was performed 72 hours post-birth, with heel blood samples collected for testing. In this study, normal reference ranges for TSH and FT4 were defined as the 2.5th to 97.5th percentiles of the general population. A TPOAb concentration ≥5.61 IU/mL was defined as positive, while the cut-off value for neonatal heel blood TSH was set at 5 mIU/L. During the initial clinical visit, maternal data were collected through interviews, including age, parity, gravidity, educational level, and smoking and alcohol consumption habits. Height and weight were measured to calculate the body mass index (BMI). Based on data from the electronic medical records system, we conducted a study to evaluate the relationship between a range of maternal and neonatal factors and neonatal heel blood TSH levels. These factors included maternal pregnancy outcomes (gestational diabetes, preeclampsia, cesarean section, placenta previa, and postpartum hemorrhage) as well as neonatal outcomes (fetal distress, preterm birth, low birth weight, macrosomia, and neonatal sex).

### Neonatal heel prick screening TSH concentration

The collection of neonatal heel blood is conducted in accordance with the standards set by the Ministry of Health’s “Technical Specifications for Neonatal Disease Screening”, Blood is collected 72 hours after birth, following at least six feedings of breast milk or formula. Two drops of capillary blood are obtained from the heel, allowing the blood to flow naturally. The drops are applied to special filter paper, saturating both sides to form two blood spots approximately 0.8 cm in diameter. The samples are air-dried at room temperature. After accurately recording the name, address, and identification number, the samples are placed in a plastic bag and stored in a refrigerator at 4°C. They are then sent to the neonatal disease screening center laboratory for testing within one week.

### Definition of pregnancy outcomes

Based on medical guidelines and literature reports, adverse pregnancy outcomes in this study were defined as follows: (1) Preeclampsia: According to ACOG guidelines, preeclampsia is defined as systolic blood pressure ≥140 mmHg or diastolic blood pressure ≥90 mmHg on two occasions at least 4 hours apart after 20 weeks of gestation in a previously normotensive woman, along with either 24-hour urinary protein excretion ≥300 mg or protein/creatinine ratio ≥0.325 (8); (2) Low Birth Weight (LBW): Neonatal birth weight <2500 g; (3) Macrosomia: Neonatal birth weight ≥4000 g; (4) Preterm birth: Defined as gestational age at delivery <37 weeks; (5) Gestational Diabetes Mellitus (GDM): Defined by a 75 g oral glucose tolerance test (OGTT) conducted between 24 and 28 weeks of gestation, with fasting plasma glucose ≥5.1 mmol/L, 1-hour plasma glucose ≥10.0 mmol/L, or 2-hour plasma glucose ≥8.5 mmol/L. (6) Postpartum Hemorrhage: Defined as blood loss >500 mL following vaginal delivery or >1000 mL following cesarean section; (7) Placenta Previa: Defined as placental extension to or covering of the cervical os at delivery; (8) Overweight and Obesity: In China, overweight is defined as a BMI >24 kg/m², while obesity is defined as a BMI >28 kg/m². (9) Fetal Distress: Defined as fetal hypoxia and/or acidosis *in utero*, manifested by abnormal fetal heart rate tracings, umbilical artery blood gas pH <7.2, meconium-stained amniotic fluid, or a 1-minute Apgar score <7 (9).

### Statistical analysis

SPSS version 21.0 (IBM Corp., Armonk, NY) and R software version 3.6.1 (R Development Core Team, July 2019; http://www.r-project.org) were used for statistical analyses. For normally distributed continuous data, values were expressed as mean ± standard deviation, and intergroup comparisons were performed using the independent samples t-test. For non-normally distributed data, values were presented as median and interquartile range, and intergroup comparisons were conducted using the non-parametric Mann-Whitney U test. Categorical data were expressed as percentages, and comparisons between groups were made using the χ² test. Univariate logistic regression with forward stepwise selection was used to analyze the relationship between maternal and neonatal pregnancy outcomes and neonatal TSH levels. Multivariate regression analysis was conducted to adjust for confounding variables that could independently influence pregnancy outcomes. Odds ratios (OR) and their 95% confidence intervals (CI) were calculated to identify risk factors and assess their impact. A forest plot was generated based on the results of the multivariate logistic regression analysis, and restricted cubic spline Cox regression analysis was performed using R software. Statistical significance was set at p<0.05 (all tests were two-sided).

## Results

### Reference ranges of TSH and FT4

**Figure 1:**
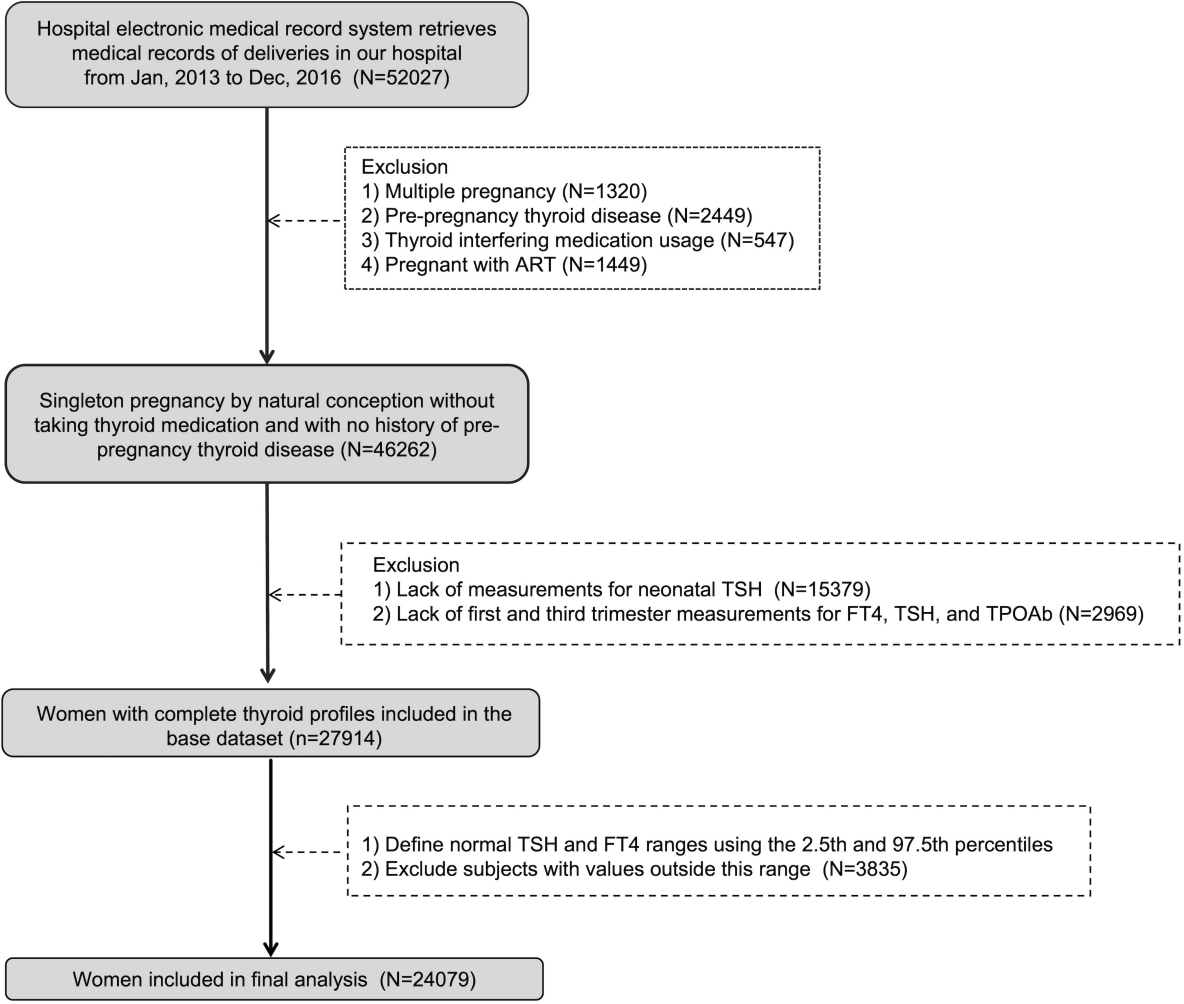
Flowchart of participants in the study.

The Endocrine Society and the American Thyroid Association recommend the use of locally determined reference ranges because the ranges of the general population do not apply to pregnant women (10). In our study, we established personalized reference ranges during the first trimester (11 to 13 weeks) and the third trimester (32 to 34 weeks) of pregnancy. In the flow chart shown in **Figure 1**, after excluding the history of thyroid disease before pregnancy and the use of drugs that affect thyroid function, we analyzed the TSH and FT4 results of naturally conceived singleton pregnant women. A total of 46262 women met the criteria, and the 2.5th percentile and 97.5 percentile were calculated as normal reference ranges of FT4 and TSH. The normal cut-off values in the first trimester (11~13 weeks) were: TSH (0.03-3.86) mIU/L; FT4 (11.6-19.8) pmol/L. While the normal cut-off values in the third trimester (32~34 weeks) were: TSH (0.38-3.67) mIU/L; FT4 (9.1-14.4) pmol/L.

### Population characteristics

A total of 24079 euthyroid singleton pregnant women were included in this study. **Figure 1** showed flowchart of participants in the study. The median neonatal TSH level in the study sample was 0.98 mIU/L (0.27-1.84 [IQR]). As shown in **Table 1**, in univariate analysis, neonatal TSH levels were significantly higher in women with advanced maternal age than in those younger than 35 years (P <0.05). TSH levels in multiparas were also significantly higher (P <0.05). However, no significant effects of maternal education level, smoking, alcohol consumption and pre-pregnancy overweight or obesity were found. **Table 1** showed the correlation between the basic characteristics of pregnant women and neonatal TSH concentrations.

**Table 1:** Univariate analysis of the association between basic maternal characteristics and neonatal TSH levels.

Basic maternal characteristics	%	Neonatal TSH level	
		Medians (IQR)	P-value*
Maternal age			**< 0.001**
age < 35y	90.0	1.01 (0.37-1.80)	
age ≥ 35y	10.0	1.11 (0.49-1.8)	
Multipara			**< 0.001**
parity < 1	85.2	1.01 (0.37-1.79)	
parity ≥ 1	14.8	1.11 (0.47-1.87)	
Education			0.170
High school and lower	28.1	1.0 (0.3-1.9)	
Bachelor	53.9	1.0 (0.3-1.8)	
Master	16.8	1.0 (0.3-1.9)	
Doctorate and higher	1.2	1.0 (0.3-1.7)	
Smoke			0.496
No	99.9	1.0 (0.3-1.9)	
Yes	0.1	1.3 (0.6-1.7)	
Alcohol use in pregnancy			/
No	100	1.0 (0.3-1.9)	
Yes	0	/	
Pre-preganancy overweight or obesity			0.675
BMI < 24kg/m2	88.6	1.03 (0.38-1.80)	
BMI ≥ 24kg/m2	11.4	1.03 (0.37-1.88)	

Bold values indicate significance at P value 0.05. * P value from Wilcoxon-Mann-Whitney test.

### Effect of maternal and neonatal outcomes on neonatal TSH concentrations

**Table 2** shows the univariate analysis of the association between neonatal TSH concentrations and perinatal outcomes. The results showed that in maternal outcomes, pre-eclampsia and cesarean section women had a significant increase in neonatal TSH level (P < 0.05), while GDM, postpartum hemorrhage, placenta previa and positive TPOAb had no effect on neonatal TSH level (P > 0.05). In addition, among the neonatal outcomes, the neonatal heel blood TSH level was significantly higher in the neonates with fetal embarrassment, male neonates, macrosomia, LBW and premature births (P < 0.05).

**Table 2:** Univariate analysis of the association between neonatal TSH concentrations and perinatal outcomes.

Perinatal outcomes	N	%	Neonatal TSH level	
			Medians (IQR)	P-value*
Maternal outcomes				
Preeclampsia				**< 0.001**
No	23662	98.3	1.01 (0.37-1.80)	
Yes	417	1.7	1.62 (1.13-2.44)	
GDM				0.275
No	21578	89.6	1.01 (0.39-1.82)	
Yes	2501	10.4	0.98 (0.33-2.75)	
Delivery mode				**< 0.001**
Vaginal delivery	13296	55.2	0.90 (0.31-1.64)	
Cesarean	10783	44.8	1.21 (0.51-2.06)	
Postpartum hemorrhage				0.091
No	23858	99.1	1.03 (0.38-1.81)	
Yes	221	0.9	1.35 (0.57-2.08)	
Placenta previa				0.260
No	23890	99.2	1.03 (0.38-1.81)	
Yes	189	0.8	1.14 (0.49-1.84)	
TPOAb				0.346
Negative	21766	90.4	1.0 (0.3-1.9)	
Positive	2313	9.6	1.0 (0.3-1.9)	
Neonatal outcomes				
Fetal distress				**< 0.001**
No	23913	97.7	1.03 (0.38-1.81)	
Yes	166	2.3	1.52 (0.77-2.63)	
Fetal sex				**< 0.001**
Male	12576	52.2	1.07 (0.40-1.87)	
Female	11503	47.8	0.99 (0.36-1.74)	
Macrosomia				**< 0.001**
No	22638	94.0	1.02 (0.38-1.80)	
Yes	1441	6.0	1.14 (0.44-1.90)	
LBW				**< 0.001**
No	23697	98.4	1.03 (0.38-1.80)	
Yes	382	1.6	1.37 (0.66-2.19)	
Premature delivery				**< 0.001**
Male	23282	96.7	1.02 (0.38-1.80)	
Female	797	3.3	1.22 (0.56-2.24)	

Bold values indicate significance at P value 0.05.

* P value from Wilcoxon-Mann-Whitney test.

**Figure 2:**
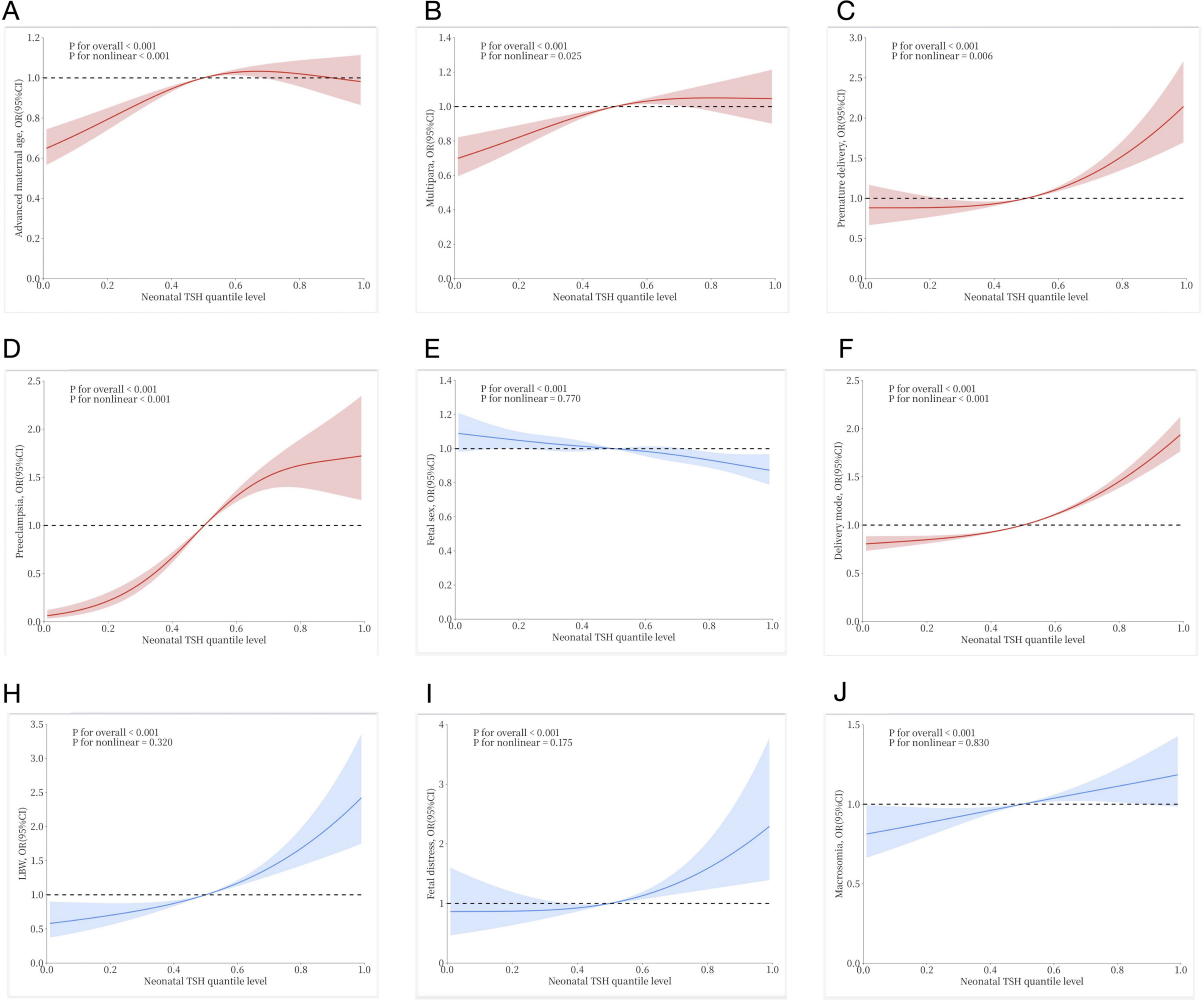
Restricted cubic spline Cox regression to show the non-linear association between neonatal TSH quantile levels and risk of various pregnancy outcomes: **(A)** Advanced maternal age; **(B)** Multipara; **(C)** Premature delivery; **(D)** Preeclampsia; **(E)** Fetal sex; **(F)** Delivery mode; **(H)** Low birth weight (LBW); **(I)** Fetal distress; **(J)** Macrosomia. The solid lines and shaded areas represent the estimated mean risk and 95% confidence intervals. Among them, red curves indicate a significant non-linear association (p for nonlinear < 0.05), while blue curves suggest no evidence of non-linearity (p for nonlinear >0.05), consistent with a linear relationship.

**Figure 2** shows the nonlinear relationship between the risk of pregnancy outcome and neonatal TSH levels as assessed by the restricted cubic spline Cox regression model. First, the overall P-values for all outcomes were less than 0.05, indicating a statistically significant association between neonatal TSH concentrations and various pregnancy outcomes. Among them, the curves drawn in blue represent cases where the overall P-value was less than 0.05 but the non-linear P-value exceeded 0.05, including E (fetal sex), H (low birth weight, LBW), I (fetal distress), and J (macrosomia). These results suggest that while neonatal TSH concentrations were significantly associated with these outcomes overall, no significant non-linear relationship was detected, implying that the associations may be closer to linear or influenced by other factors. In contrast, the curves drawn in red represent cases where both the overall and non-linear P-values were less than 0.05, including A (advanced maternal age), B (multipara), C (preterm delivery), D (preeclampsia), and F (delivery mode). These results indicate that neonatal TSH concentrations not only exhibited a significant overall association with these pregnancy outcomes but also displayed a non-linear relationship.

### Relationship between pregnancy outcome and neonatal TSH elevation

**Figure 3:**
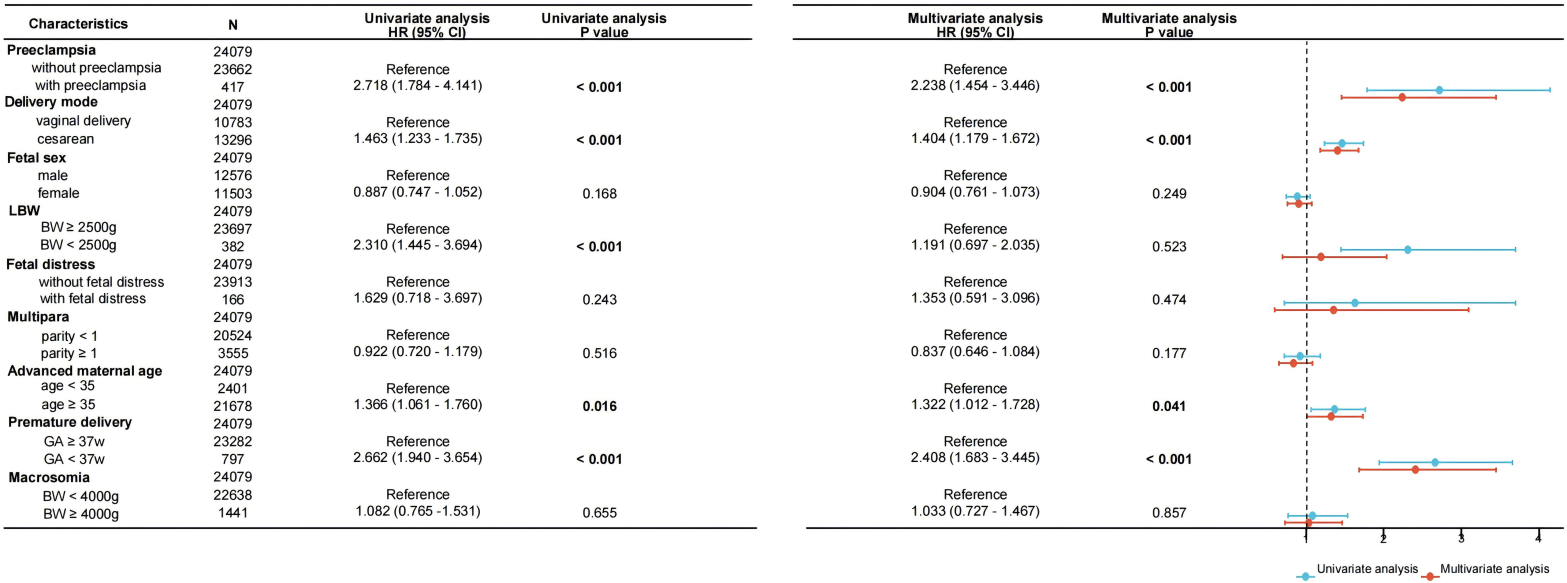
Forest plot of univariate and multivariate logistic regression analyses for the association between neonatal TSH concentrations and perinatal outcomes. Data are presented as odds ratios per standard deviation change in the respective variable. BW, birth weight; GA, gestational age.

To further investigate the association between pregnancy outcome and neonatal TSH elevation, we used univariate and multivariate logistic regression analyses with correction for possible confounding factors. Using 5 mIU/L as the threshold, we converted neonatal TSH levels into a binary variable, and the results of the logistic regression models are shown in **Figure 3**. Univariate regression analysis showed that preeclampsia, cesarean section, LBW, advanced maternal age, and preterm birth were significantly associated with increased neonatal TSH levels (P<0.05). After adjusting for confounding factors such as neonatal sex, LBW, fetal distress, multiparity, and macrosomia, the following pregnancy outcomes remained strongly associated with neonatal TSH elevation (P<0.05): preeclampsia (OR=2.238, 95% CI 1.454 ~ 3.446), cesarean section (OR=1.404, 95% CI 1.179 ~ 1.672), advanced maternal age (OR=1.322, 95% CI 1.012 ~ 1.728), and preterm birth (OR=2.408, 95% CI 1.683 ~ 3.445).

## Discussion

Most countries accept the use of TSH screening via heel prick or umbilical cord blood as a method for identifying congenital hypothyroidism (CH) (11). Samples can be collected from umbilical cord blood at delivery or obtained through heel prick on days 2–3 after birth. Both collection methods are considered suitable for screening purposes (12). Compared to umbilical cord blood sampling, heel blood sampling better reflects the thyroid function status of newborns after birth. Additionally, due to its simplicity and convenience, heel blood sampling has become the routine method for screening congenital hypothyroidism and is widely implemented around the world. Since 1994, the World Health Organization (WHO) has proposed that blood spot TSH screening can serve as a biomarker for assessing population iodine status (13). When the proportion of newborns with blood spot TSH concentrations exceeding 5 mIU/L is less than 3%, it is considered that the iodine intake of that population is adequate. Although advancements in measurement technology now allow for precise quantification to several decimal places, the WHO continues to use the 5 mIU/L standard to facilitate global data comparisons. Based on this criterion, 5 mIU/L has become a commonly used threshold in neonatal TSH screening. In this study, we also utilized 5 mIU/L as the cutoff for abnormal neonatal TSH levels, converting TSH into a categorical variable for inclusion in both univariate and multivariate logistic regression models.

Before 11 weeks of gestation, the fetal thyroid lacks the ability to concentrate iodine and synthesize thyroid hormones. During this period, the fetus relies entirely on maternal thyroid hormones for its growth and development. After 11 weeks, as the fetal hypothalamus and pituitary begin to develop, the thyroid gradually becomes functional, with full development occurring by 20 weeks. Thus, the maternal thyroid function before this time directly affects fetal growth and is closely associated with neonatal TSH levels (14). Studies have shown a positive correlation between maternal TSH levels and neonatal TSH (15, 16). Those mothers with hypothyroidism were more likely to exhibit elevated TSH levels during thyroid screening (17). Korevaar et al. (16) conducted a retrospective clinical analysis, demonstrating that maternal thyroid hormone and TSH levels may be associated with offspring TSH and T4 levels. When pregnant women have autoimmune thyroid disease, TSH receptor antibodies can cross the placenta, potentially leading to fetal hyperthyroidism or hypothyroidism (1). Additionally, pregnant women with hyperthyroidism who receive excessive antithyroid medication may give birth to infants with hypothyroidism. Maternal medical conditions (7) and a history of hypothyroidism (18) have also been associated with significantly elevated cord blood TSH levels. Moreover, some clinical studies have found that neonates conceived by ART (assisted reproductive technologies) have higher cord blood TSH levels compared to those without ART (5). To better explore the relationship between pregnancy outcomes and neonatal TSH levels, this study excluded participants with abnormal thyroid function tests (FT4 and TSH) in early or late pregnancy, those taking thyroid medications, individuals with chronic medical conditions, twins, and those conceived through ART. The statistical analysis also included an evaluation of the impact of TPOAb on neonatal TSH levels.

Research has shown that perinatal thyroid function is dynamic, with the secretion and metabolism of thyroid hormones undergoing changes after birth to enable the infant to adapt to the constantly evolving demands of postnatal life (19). Several studies indicate that numerous factors can influence maternal, fetal, and neonatal TSH levels (5). Additionally, at birth, newborns experience elevated thyroid hormone levels due to physiological factors such as thermoregulation and the absorption of amniotic fluid, which play a critical role in their postnatal adaptation (5, 20, 21). Previous studies have demonstrated that perinatal factors such as maternal BMI, age, gestational age, mode of delivery, parity, and newborn birth weight may impact neonatal TSH levels. Clinically, it is essential to consider these perinatal factors when interpreting neonatal thyroid function accurately. However, many past studies were conducted with small sample sizes and yielded inconsistent results. They often focused on one or two variables, without fully accounting for the complexity of these factors, potentially leading to erroneous conclusions. Conducting multivariable analyses to account for highly correlated maternal, delivery, and infant factors presents certain challenges. In this study, based on a large retrospective cohort, we performed both univariate and multivariate analyses to examine the impact of various perinatal maternal and fetal factors on neonatal heel blood TSH levels. This approach aims to provide further evidence for optimizing screening standards and to offer a more objective interpretation of neonatal TSH screening results.

It is generally believed that preterm infants and those with low birth weight (LBW) tend to have higher TSH levels. Studies have shown that neonates with lower gestational age often exhibit higher TSH levels in both umbilical cord blood and heel prick samples, while their T4 levels are typically lower (5). Korada et al. highlighted a significant inverse relationship between neonatal TSH levels and birth weight (22). Similarly, Herbstman et al. found that as gestational age increases, cord blood TSH levels show a marked decrease (5, 23). Other studies have also indicated that reductions in gestational age and birth weight are significantly associated with elevated TSH levels (24). Our study aligns with these previous results, showing that in univariate analyses, both LBW and preterm birth were associated with higher neonatal TSH levels. However, after adjusting for various confounding factors in multivariate analysis, only preterm birth remained closely linked to elevated neonatal TSH levels. There have been conflicting reports, though. For instance, Dalmazi et al., in a retrospective study, found that small-for-gestational-age full-term infants had higher TSH levels compared to normal full-term infants (25). The mechanisms behind elevated TSH levels in preterm or LBW infants remain unclear, but immaturity of the hypothalamic-pituitary-thyroid (HPT) axis and impaired regulatory capacity are thought to play key roles (5, 8, 26). Preterm infants, despite their developmental immaturity, must adapt to the harsh extrauterine environment. Their impaired HPT axis and insufficient thyroid hormone secretion may contribute to elevated TSH levels. Additionally, preterm birth leads to the abrupt cessation of maternal thyroid hormone transfer via the placenta, coupled with the infant’s immature HPT axis, resulting in low T4 levels, a condition often referred to as transient hypothyroxinemia of prematurity (9). This condition is typically short-lived, and as the HPT axis matures, TSH and T4 levels usually return to normal ranges. Overall, both low birth weight and preterm birth significantly influence neonatal TSH levels, and these factors may have lasting effects on thyroid function.

Current research has yet to conclusively determine whether the mode of delivery affects neonatal thyroid hormone levels. Several studies suggest that cesarean delivery may lead to elevated TSH levels in neonatal blood samples (24, 27). For example, Ryckman and Wang et al. found that neonates born via vaginal delivery had lower TSH levels (15, 24). Our findings align with these studies, showing that neonates born to mothers who underwent cesarean section had higher heel blood TSH levels. However, some researchers argue that vaginal delivery may lead to higher TSH levels in umbilical cord blood (5, 28, 29). These studies highlight the significant impact of the birth process on the hypothalamic-pituitary-thyroid (HPT) axis at birth. The changes in neonatal TSH levels following birth are a crucial physiological response to adapting to the external environment. This shift primarily stems from the newborn’s transition from the warm intrauterine environment to the relatively colder extrauterine world (30). Neonates delivered by cesarean section tend to have lower axillary and skin temperatures compared to those born via vaginal delivery. Cold exposure stimulates the newborn’s hypothalamus to release thyrotropin-releasing hormone (TRH), which in turn promotes the pituitary gland to secrete TSH, resulting in elevated TSH levels. This alpha-adrenergic stimulation following cold stress is one of the reasons for the TSH increasing in newborns (31). Hence, the lower body temperature of cesarean section neonates may trigger an increase in TSH levels. Additionally, anesthesia or surgical stress may also contribute to elevated TSH levels (32). Notably, it is currently believed that differences in thyroid hormone levels among newborns born via different delivery modes are temporary (33). All newborns tend to have similar thyroid hormone levels by the 24th day after birth. This suggests that the impact of delivery mode on neonatal thyroid hormone status may be short-term.

Herbstman et al. found evidence of an association between preeclampsia and lower cord blood T4 levels (5). Previous reports (34) suggest that maternal conditions affecting placental dynamics may also impact neonatal thyroid function. Chen et al. demonstrated a significant correlation between preeclampsia and elevated neonatal TSH levels (7), and a systematic review (33) confirmed that neonates born to mothers with preeclampsia generally have higher cord blood TSH levels. Similarly, Ryckman et al. found elevated TSH levels in neonates born to mothers with preeclampsia, likely linked to placental insufficiency or fetal hypoxia (24). Our findings align with these observations, as we also noted higher neonatal TSH levels in infants born to preeclamptic mothers. However, other studies have reported contradictory results (5, 24, 35). It is believed that elevated maternal systolic blood pressure during pregnancy can lead to persistent placental hypoperfusion, resulting in fetal ischemia and hypoxia. Hypoxia and acidosis may trigger redistribution of fetal blood flow to increase cerebral perfusion, thereby stimulating TSH secretion (36) and contributing to neonatal thyroid dysfunction. On the other hand, decreased serum thyroid hormone levels may reduce oxygen consumption in brain tissue, offering neuroprotective effects. In summary, preeclampsia may be associated with elevated neonatal TSH levels, reflecting complex placental dysfunction and fetal developmental issues. Further research is needed to elucidate the underlying mechanisms and determine optimal management strategies.

Several studies have suggested an association between maternal age and neonatal thyroid function. A study from China reported that advanced maternal age is linked to lower FT3 levels in cord blood (29). Similarly, research by Rezaeian found that advanced maternal age increases the risk of neonatal congenital hypothyroidism (CH). A study conducted in rural India further demonstrated a steady increase in cord blood TSH concentrations with advancing maternal age (18). Our findings align with these studies, indicating that neonates born to mothers with advanced age tend to have higher TSH levels in heel-prick blood samples. However, some studies did not find a significant association between maternal age and neonatal TSH concentrations (5, 34). The potential mechanisms underlying this phenomenon may relate to changes in maternal thyroid function and iodine status. Research suggests that with increasing maternal age, the activity of type 2 deiodinase (D2) declines, potentially leading to lower fT3 levels in cord blood (29). In contrast, type 3 deiodinase (D3) expression is upregulated in cases of hypoxia and maternal aging, which could be linked to an increased risk of intrauterine hypoxia (37). Changes in the expression of D2 or D3 are associated with variations in neonatal TSH levels. Clinical studies also indicate that advanced maternal age increases the risk of CH in offspring (38), potentially due to the physiological decline and impaired iodine metabolism observed in older mothers.

## Conclusion

Neonatal TSH levels are associated with various maternal and neonatal factors, including advanced maternal age, preeclampsia, cesarean delivery, and preterm birth. These factors can all contribute to higher levels of TSH in newborn heel prick blood samples. Therefore, when evaluating TSH levels in newborn screening, it is crucial to consider these perinatal conditions to make more accurate assessments.

## Strengths and limitations

Previous studies evaluating the relationship between various perinatal factors and neonatal TSH levels typically included only a small number of subjects. Moreover, most of these studies (39) used univariate analyses to assess the influence of perinatal factors on neonatal TSH levels, which can introduce potential bias as confounding factors, such as maternal TPOAb, were not adequately considered. The strengths of our study lie in the stringent inclusion criteria, excluding subjects with abnormal thyroid function indicators during pregnancy, those taking thyroid medication, those with pre-pregnancy thyroid disease, multiple pregnancy, and pregnancies conceived by ART, all of which could potentially affect offspring TSH levels. We conducted a comprehensive evaluation of key maternal and neonatal factors influencing neonatal TSH, with a large sample size providing robust data. Additionally, the use of multivariable analysis is another strength, as it allowed us to account for the true impact of the variables analyzed on neonatal TSH levels. However, the limitations of our research include its reliance on single-center data and its observational design, which may lead to selection bias and preclude definitive conclusions about causality. Secondly, maternal iodine nutritional status was not assessed in this study. As iodine is a key nutrient affecting thyroid function, inadequate or excessive iodine intake during pregnancy may influence neonatal TSH levels. Therefore, future large-scale multicenter studies incorporating iodine status and other relevant biological variables are needed to further clarify the impact of maternal and neonatal factors during pregnancy on neonatal TSH levels.

## Data Availability

The raw data supporting the conclusions of this article will be made available by the authors, without undue reservation.
